# Gall Passage Diagnosis

**Published:** 1917-03

**Authors:** W. C. Calvert

**Affiliations:** Mineola, Texas


### Gall Passage Diagnosis.

BY W. C. CALVERT, M. D., MINEOLA, TEXAS.

A broad survey of the gall passage diseases shows three general divisions, namely: Inflammatory processes, new growths, and accidental conditions following either the inflammatory or the new growth changes. This paper will consider only the inflammatory processes and the incidental accidental conditions.

It may be stated that the diagnosis of gall passage conditions must depend upon the general symptomology presented by the inflammation with certain symptoms due to special locations. But, with a local anatomical structure surrounded by so many important organs, the local coloring given to the general symptomology may be most confusing, indeed, oftentimes wholly misleading. Again, the ever present reflex symptoms or manifestations complicate still further efforts at diagnosis. Owing to the extensive scope of this paper, a general outline only can be given with the result that many interesting and important phases of the subject must be brought out in the discussion.

The inflammatory picture includes: Inflammation of the mucous membrane of the small intestines; of the common cystis and hepatic ducts; of the gall bladdeer; of the lungs, pleura, and peritoneum; of the appendix; and of the large intestines. The virulence of the infection in any of these parts is an important factor. The infection may be mild and slow of development, virulent and rapid, or at any point of severity in the long scale between these two extremes. In cases of mild infection, the reaction may be so slight as to escape detection,—no fever, no leucocytosis, and the minimum or no local disturbance. Although without symptoms, such an infection may continue until the cumulative injury and the changes from the normal may produce varying degrees of discomfort, even localized pain over the gall bladder regions, or some degree of reflex disturbance, such as stomach trouble. Eventually, they may lead to the production of gallstones or to other accidents, such as ulcer formations followed by scar formation or rupture.

On the other hand, a virulent infection may produce a pronounced classical clinical picture of, say, gall bladder empyema within a few hours.

*Inflammation.*—The varied manifestations of inflammation in the bile-passages are most difficult of description on account of the almost infinite number of clinical pictures that they present. We find, first, catarrhal jaundice. This condition is caused by an extension of inflammation from the intestines, producing swelling of the mucous membrane and blocking of the intestinal end of the common duct. This leads to obstruction of the common duct, enlargement of the liver and the gall bladder, and to jaundice. Fever is present, pain is usually absent, and the conditions ends, without complication, within a few days or a week.

The diagnostician of catarrhal jaundice depends, as a rule, upon the age of the patient. The disease occurs most frequently in the young. The presence of gastro-intestinal disturbance before the onset of jaundice, the presence of fever, and the absence of other signs and symptoms save jaundice are indicative of catarrhal jaundice.

Differential diagnosis must consider the infection of gallstones. Here we have the history: Pains, fever, chills and sweats. These symptoms serve to differentiate this conditions from Weil's disease, characterized by acute onset, fever and splenic enlargement, from acute yellow atrophy characterized by failure of the jaundice to clear up. Grave cholemic symptoms, rapid diminution in the size of the liver, and aminoacids in the urine; and from carcinoma compressing the ducts choledochus characterized by the age limit, long duration, emaciation, and cachexia.

Second. Extension of the inflammation through the common duct to the gall bladder, commonly known as "cholecystitis." Here many gradations of inflammation occur, both as to time and to severity. The inflammation may be mild and of a transient nature. In this event, the lining membrane of the gall bladder is involved, and some thickening of the bladder wall follaws. At this time there may be no distinct reaction of inflammation. The patient may not even notice any change from the normal. On the other hand, some illy-defined feelings of discomfort may be noted amounting only to a mild indisposition. Again, the slight disturbance may cause some mild symptoms of indigestion. Not enough, however, to impress itself distinctly upon the patient.

Such a mild inflammatory process may recur, repeating the illy-defined symptoms, or producing no symptoms whatsoever. As time passes, the effects of mild inflammation gradually accumulates until a degree of pathological change in the gall bladder is reached sufficient to produce some mild symptoms. But years may elapse from the first infection to the development of symptoms. The chief diagnostic symptoms are:

(a) History of repeated periods of illy-defined indisposition, following some infectious disease.

(b) Discomfort, sometimes amounting to pain elicited by pressure over the gall bladder, especially on deep inspiration.

(c) A distinct, although slight, gastric upset.

In more severe cases, there is pain and tenderness in the region of the gall bladder, rigidity of the right upper rectus, nausea and vomiting, and sometimes an enlarged and tender gall bladder. The tumor representing the distended gall bladder moves with respiration. Leucocytosis is rarely above 15,000.

Differentiation diagnosis must consider acute appendicitis and acute peritonitis due to perforation of a gastric or a duodenal abscess.

All of these symptoms are very mild, and they mark the first recognizable gall bladder attack. Fever may or may not be present. This upset may last from a few hours to a number of days and end in recovery. As time passes, recurring attacks develop, varying greatly in severity, often gradually increasing in duration, severity and in the degree of fever. In such a case, the diagnosis is usually very easily made. The diagnostic points are: Recurring attacks of indigestion, or rather stomach disturbance associated with some tenderness over the gall bladder and with varying degrees of fever.

Too often, however, such a regular course of development does not follow. The inflammatory process may, during any attack, become more severe, involving greatly the wall of the gall bladder. Now the discomfort develops to real pain, which may be localized or radiate to the right shoulder. At times the pain may be severe—cutting. Again, it may be referred to any portion of the abdomen, most commonly to the right half and toward the navel. This referred pain is the most significant symptom to keep in mind, else the gall bladder may be completely overlooked. Not uncommonly, the pain may simulate that of appendicitis, or vice versa. Pressure over the gall bladder elicits pain. The digestive disturbances are marked, oftentimes associated with vomiting and occasionally with diarrhea. Fever is usually present. The leucocytes may be slightly increased. These attacks continue for from a few hours to a number of days, and end in recovery. Here the picture may change rapidly. It may involve the walls of the gall bladder, ulcerate rupture or pass to the capillary bile passages.

Thus far, the anatomical changes in the gall bladder usually are not sufficient in degree to produce enough shadow in the X-ray dense to be of diagnostic value. Gallstones may or may not be present in such a bladder. If present, the additional symptoms may be observed. The inflammatory process in the gall bladder may extend as follows:

(1) To the cystic duct causing hydrops or empyema of the gall bladder.

(2) To the bile capillaries°chlogngitis°giving rise to chills, fever and sweats.

Or the gall bladder inflammation may cause:

(1) Blocking of the common duct causing chronic jaundice.

(2) Perforation of the gall bladder or duct.

(3) Perforation of the intestines.

Chronic cholecystitis gives the same symptoms as do gallstones.

The inflammatory process may pass beyond the walls of the gall bladder to involve the peritoneum and the omentum. As a rule, when the peritoneum is involved the pain is more intense, and muscular rigidity may be expected. At this time, only light pressure is required to elicit a rather severe pain. The digestive symptoms are well marked, and the fever may reach 102°, even higher. Slight leucocytosis is present.

After the peritoneum becomes involved the omentum often attaches itself to the inflamed parts, and peritoneal adhesions form. From this point on, the condition is more complicated and more difficult of diagnosis. The bulk of the adhesions and the rolling up of the omentum may produce a mass most difficult to interpret, especially in the presence of a thick abdominal wall. Adhesions may displace the pylorus and portions of the intestines, thus developing pressure on the common duct or the intestines. This condition may obstruct the common duct and cause jaundice, or it may obstruct the intestines. In this event, the symptom of intestinal obstruction may predominate and completely obscure the gall bladder condition.

If the adhesions are extensive, marked deformity in the pyloris, jejunum and the hypatic flexure of the colon may occur. These deformities may be distinctly shown by means of the barium meal under the X-ray. Primary lesions in the pyloris may produce similar deformities, but, as a rule, in pyloric lesions the adhesions are not so extensive as in gall bladder infections, and the lesions in the pylorus may be seen in the X-ray, whereas, in gall bladder adhesions the pylorus presented no lesions, save the deformity. The cap may be greatly deformed. Obstruction in the intestines are very clearly shown, either by the slowness of the passage of the barium through the intestines, or by its failure to pass at all. If, perchance, gallstones are present, they might be seen in the X-ray.

A typical symptom complex would produce, on physical examination: A mass or sense of resistance or thickening of parts, muscular rigidity, intense pain, leucocytosis rarely about 15,000, marked disturbance of digestion, and fever to 103°. Jaundice is also present if obstruction is produced by pressure or adhesions. Intestinal obstructions also may complicate the picture.

Case 1.—Woman. A few light attacks of indigestion with slight indigestion between these attacks for fifteen years. Present illness began rather suddenly; had continued for three or four days. Temperature, 102°. Sensation on palpation of a mass in the upper right abdomen. Muscular rigidity. Very tender on pressure. Not much continuous pain. No vomiting. Some nausea. No appetite. Jaundice. Otherwise, negative. Possible diagnosis: New growth, or inflammation of the gall bladder. Final diagnosis: Infection of the gall bladder, peritonitis and obstruction. Operation. Omentum adhered to the region about the gall bladder. Numerous adhesions. Gall bladder completely destroyed. Gangrene.

After the infection passes beyond the walls of the gall bladder the subsequent history depends upon the virulency of the invading organism and the region invaded, or the direction in which the infection travels. The local peritonitis may serve as a closing chapter. But, in the event of a low virulency of the invading organism, or the successful walling off of the peritoneal cavity, very little local infection may develop; sometimes, not enough to attract attention. In such cases the infection travels along the path of least resistance to form an abscess in some neighboring structure. These cases are most difficult of detection; in fact, are oftentimes impossible. The symptoms are those arising from the infected organs, such as the heart, the lungs, the pleura, or the distant portions of the abdomen. On the other hand, in primary infections affecting the heart, the lungs, the pleura, the stomach and the distant portions of the abdomen, the symptoms may so closely resemble gall bladder conditions as to obscure completely the real situation.

Case 2.—Man. History negative. Sick four days. Pain in upper right abdomen. Temperature, 100°. Marked muscular rigidity. Pain on pressure. Region of appendix tender. Some disturbance of the stomach. Liver dullness to the fourth rib. Rales in lower right lung. Considerable dullness in lower side and back of the right side. Leucocyte count, 25,000. The muscular rigidity, tenderness of gall bladder and stomach symptoms strongly suggested an infections about the gall bladder. The high liver dullness, rales, dullness in side and back, and the high leucocyte count suggested an infection of the pleural cavity—possibly, a diaphragmatic abscess. A small amount of clear fluid was withdrawn from pleural cavity. The subsequent history of this case revealed an infection of both the gall bladder and the pleural cavity. The infection could easily have extended from the region of the gall bladder to the pleural cavity.

Case 3.—Young man. Sick about 10 days. History of acute dysentery during previous summer. Fever, 104°. Sweats and chills. A very little loss in weight. Rigidity over the region of the gall bladder. Very painful. Liver large extending to the crest of the ileum. Rales and dullness in lower right lung. Otherwise, heart and lungs negative. Leucocytes of 30,000. Slightly jaundiced. The history as obtained was negative, except the family history of tuberculosis. X-ray revealed heart and lungs normal, large liver; otherwise negative. Diagnosis was obscure. The presence of pus was evident. It was thought to be either in the gall passages, liver, or about the diaphragm. An exploratory operation revealed liver and bile passages normal. A deep puncture found about one and one-half pints of pus above the diaphragm. Diagnosis: Diaphragmatic abscess. Autopsy one week later revealed malignant endocarditis.

When the infection extends through cystic ducts to the bile passages in the liver, various clinical pictures are presented. These range from a simple catarrhal change to an active inflammation, often advancing to pus formation, liver abscess and septicemia. When the inflammation is active the reaction to infection is severe. The liver is enlarged and usually tender; the gall bladder is usually enlarged; pain, however, is not a prominent symptom; jaundice is common; high fever, chills and sweats are frequent, with the leucocytosis high and the spleen enlarged. This condition is usually associated with an infection of the gall bladder, especially when stones are present, or of the common duct, possibly of both. Fistulous communications may be established with the duodenum, colon, pleura or lung.

Thus far, the catarrhal and destructive inflammatory lesions have been considered. Empiema of the gall bladder may develop from the foregoing lesions at any time. Collection of pus in the gall bladder may or may not produce a large gall bladder. The condition of its walls at the time of infestion must determine their elasticity. The symptoms rapidly develop and are severe. Paroxymal pains in the right side of the abdomen, especially in the region of the gall bladder, followed by nausea, vomiting, rapid pulse, rise in temperature, distention of the abdomen, abdominal tenderness anywhere, moderate leucoyctosis and prostration are the most distinctive symptoms. The diagnosis is uncertain. The pain or tenderness may be anywhere in the abdominal distention so often obscures the field. Appendicitis, gastric conditions, intestinal obstructions may be suspended. Unless the condition is typical, an exploratory operation alone will reveal the condition.

Obstruction of the cystic duct, either by gall stones or by inflammatory processes, usually leads to distension of the gall gladder. The side of the bladder may vary from about normal to an enormous cyst-like structurie, which at times has been mistaken for ovarian cysts. Such a bladder may be distinctly felt through the abdominal walls. Its contents may vary from a thin, clear, mucoid, fluid to pus. The diagnosis must depend upon the degree of infection present in the distended gall bladder and, in the event of the absence of infection, upon the physical form of the bladder. There is present no jaundice, unless an obstructing stone protrudes into and obstructs the common duct or the extension of the inflammatory processes causes destruction and blocking of the common duct.

### GALLSTONES.

Gallstones may remain indefinitely in the gall bladder without producing symptoms. On the other hand, according to Moynihan, the early symptoms of gall stones are: A sense of fullness, weight and oppression in the epigastrium, a catch in the breath, a feeling of faintness, nausea or vomiting and a chilliness after eating.

In the event the gall stones become dislodged to enter the cystic or the common duct, if too large to pass through these structures, biliary colic results. The symptoms of this colic are: intense colicky pains in the upper abdomen, radiating to the navel or to the thoracic regions, a chill, and fever. In the event the pains are not colicky, they are constant and dragging, increasing when food is taken. It may require from a few minutes to a number of days for the stone to pass through the duct. During the severity of the attack, the patient may be covered with perspiration and be prostrated. Gastric symptoms are usually present.

DIAGNOSIS.

The history of previous attacks is most important. In nervous women pseudo-biliary colic with pain on the right side, enlarged liver without inflammation occurs. One may suspect stomach ulcer. If the typical picture of colic with jaundice is present, the diagnosis offers no difficulty.

If infection is added to the stones and gall bladder symptoms, a common occurrence, the picture of cholecystitis, previously described, is produced.

If the dislodged stone cannot pass through the common duct, it becomes lodged, usually at the intestinal end or in the diverticulum or vater. The stone may completely close the common duct—a permanent occlusion—in which case the jaundice is deep and permanent. The pain, the history of biliary colic and the absence of an enlarged gall bladder help to differentiate this condition from new growths. Incomplete obstructions, by a series of stones or by a stone in the diverticulum or vater may continue without symptom, but as a rule are associated with varying degree of infection and a considerable dilatation of the gall passages, but without dilatation of the gall bladder. The chief symptoms are the continuous or periodical presence of bile in the feces, a variation in the intensity or a persistent jaundice, normal size or only sligtly enlarged liver, no distension of the gall bladder, and enlargement of the spleen.

The symptoms of a ball-valve stone are: Chills, fever and sweating; varying degrees of jaundice increasing after each attack, pains in the region of the liver with disturbances of the stomach. The fever may go to 103, the chills may occur at regular or irregular intervals; the condition may last over a long period of time, and infection may be added to the picture at any time.

Gallstone symptoms must be differentiated from renal colic, intestinal colic, and lead colic. The early symptoms associated with indigestion must be differentiated from gastric ulcer, duodenal ulcer, and carcinoma of the gall bladder.

### OBSTRUCTION OF THE BILE DUCTS.

At first the liver is enlarged; later it may be much reduced in size. Intermittent fever, so usual in common duct stone is often present.

*Diagnosis.*—A history of colic, varying degrees of jaundice paroxymal pains, all point to gallstones. The gall bladder is usually enlarged. In obstructions of the common duct, except in the case of gall stones (Courvoisier's Law) progressive enlargement of the liver with jaundice and a moderate continued fever is usual in the common duct disturbances. In obstructions from the lymphatic glands, the general glandular system should be estimated, especially those above the clavical.

During the past few years an enormous amount of work has been done in developing and testing methods for determining the fuctional activity of the liver. All of these tests are based on one or more of the various metabol functions of the liver and usually depends upon some actual destruction or interference with the liver substances. As a differential, then, they are in reality more important in the diseases affecting the liver substances than in the diseases involving the bile passages. Like all functional tests, they are at best relative and as a rule of little or no value, unless they are often repeated, each as a check upon the others. Such repetition consumes considerable time, the amount of time necessary considered in connection with their relative value serves further to lessen their practical value as a differential diagnostic agent. However, in some instances where there is inflammation of the bile passages, one or more of the functional tests may render aid. Of the various tests in use, the excretion of phenoltetrachlor phthalien is perhaps most available. This chemical is injected intra-venously, then recovered in the feces and quantitatively determined by the same method as that used for the phthelien test in the urine. Its usefulness in the infectious diseases of the bile passages is of a negative character. If the amount excreted is normal, it is a fair indication that the smaller bile capillaries and common ducts are at least relatively free and that the symptoms present in the case are in the main arising from the gall bladder. Where jaundice is present with ill-defined symptoms, it may serve to differentiate obstructions of the common duct from hemolytic jaundice. The other functional tests are too indefinite in their results and too expensive in time to justify their use.

### CONCLUSION.

In conclusion, it may be noted that nowhere in medicine is an accurate detailed history of more importance in diagnosis than in gall-bladder diseases. Of especial importance is the history of repeated attacks of indigestion, associated with pains over the gall-bladder region and jaundice. Clay-colored stools due to the absence of bile is another confirmatory symptom. The urinary findings are of the minimum importance, save the presence of amino-acids. Cholemic symptoms are usually absent save when the liver substances are involved. The pancreas must always be considered in cases showing obstruction of the end of the duct. Carcinoma is a most common lesion. Chronic jauncreatitis is often associated with the common duct obstruction and may become an important factor in the obstruction. Enlargement of the spleen is usually associated with enlargement of the liver. Carcinoma of the liver and fatty liver are the exception.

